# Evolutionary rates and centrality in the yeast gene regulatory network

**DOI:** 10.1186/gb-2009-10-4-r35

**Published:** 2009-04-09

**Authors:** Richard Jovelin, Patrick C Phillips

**Affiliations:** 1 Center for Ecology and Evolutionary Biology, 5289 University of Oregon, Eugene, OR 97403, USA

## Abstract

**Background:**

Transcription factors play a fundamental role in regulating physiological responses and developmental processes. Here we examine the evolution of the yeast transcription factors in the context of the structure of the gene regulatory network.

**Results:**

In contrast to previous results for the protein-protein interaction and metabolic networks, we find that the position of a gene within the transcription network affects the rate of protein evolution such that more central transcription factors tend to evolve faster. Centrality is also positively correlated with expression variability, suggesting that the higher rate of divergence among central transcription factors may be due to their role in controlling information flow and may be the result of adaptation to changing environmental conditions. Alternatively, more central transcription factors could be more buffered against environmental perturbations and, therefore, less subject to strong purifying selection. Importantly, the relationship between centrality and evolutionary rates is independent of expression level, expression variability and gene essentiality.

**Conclusions:**

Our analysis of the transcription network highlights the role of network structure on protein evolutionary rate. Further, the effect of network centrality on nucleotide divergence is different among the metabolic, protein-protein and transcriptional networks, suggesting that the effect of gene position is dependant on the function of the specific network under study. A better understanding of how these three cellular networks interact with one another may be needed to fully examine the impact of network structure on the function and evolution of biological systems.

## Abstract 2

Yeast transcription factors that are more central in the transcription network tend to evolve more quickly.

## Background

Understanding of the function and evolution of any specific gene or protein requires knowledge of the context in which that gene operates, because change in any single component of a complex system can have ramifications for all other components. This system-orientated view, largely enabled by the omics revolution, has sparked increasing interest in the investigation of biological networks and has yielded promising results in the understanding of cellular [1], developmental [2] and ecological [3] processes. A major challenge within this area is to determine how the various parts of a system interact in order for the system as a whole to function. With a more global understanding of system function in hand, a larger question then emerges: in what ways does the structure of the network influence the evolution of the components of that network? For example, in the yeast protein-protein interaction (PPI) and metabolic networks, central and highly connected proteins tend to evolve more slowly than peripheral genes [4-7]. Is this a global feature of all such networks, or does the specific function of a given network have a strong influence on its evolutionary properties? Here, we address these questions by analyzing the evolution of the yeast transcription factors in the context of the structure of the transcriptional regulatory network.

The premise that biological systems are more than the sum of their parts implies that such systems possess emergent properties that cannot be captured by a purely reductionist approach. For a network, one such emergent property is its topology. Comparisons of entirely different types of networks, including social, technological and biological networks, have revealed intriguing shared topological properties, such as an overall hierarchical organization, similar node-degree distributions, and a tendency toward a small-world structure in which most nodes are connected by only a few other intervening nodes [1]. The observation that both metabolic and PPI networks display approximately scale-free topologies, with a few highly connected nodes and a majority of nodes with only a few connections, leads to the proposal that network structure may be the result of selection, perhaps as a means of providing mutational robustness [8]. This hypothesis remains uncertain, however, because networks with node connectivity following a power-law distribution can be assembled without natural selection [9] and because natural selection is very weak on second order network properties such as robustness [10]. Further, networks with similar power-law distributions can have different fine-scale architectures, which may be functionally important [11].

In this study we examine the evolution of the yeast transcription factors and ask whether fine differences in network structure and function lead to different evolutionary impacts on the elements of those networks. Gene regulatory networks are of particular interest because they allow the cell to modify its physiology, cycle and shape in response to environmental or developmental demands [12]. Metabolic and gene regulatory networks have a different level of complexity than PPI networks because they are directed and explicitly model the flow of information passing through the nodes. Moreover, even though all three cellular networks are characterized by having a small number of highly connected nodes, these networks differ in their node-degree distribution [1]. The yeast transcription regulatory network consists of a mixed scale-free and exponential topology: only the number of target genes follows a power-law distribution whereas the number of regulators is exponential [13]. These structural and functional differences may result in different effects on the evolution of network components. For instance, underlying the power-law distribution of target genes is a distributed architecture that may cause the apparent independence between connectivity and the retention of regulatory proteins across genomes [14].

Overall, we show that network structure does indeed lead to different evolutionary dynamics that depends more specifically on the overall function of the network. Therefore, understanding the relationship between network structure and the evolution of network components will depend on a deeper knowledge of gene function.

## Results

### Central transcription factors tend to evolve faster

We obtained node statistics, specifically the number of regulatory inputs (in-degree, *k*_*in*_), the number of target genes (out-degree, *k*_*out*_), and betweenness, measuring the centrality of a gene in the network, from two separately derived representations of the yeast transcriptional network. The first dataset (YTN1) [15] includes 286 transcription factors, 3,369 target genes and 8,372 regulatory interactions. The second dataset (YTN2) [14] includes 157 transcription factors, 4,410 target genes and 12,873 regulatory interactions. Only transcription factors clearly identified as orthologs in the yeast genome database (*Saccharomyces *Genome Database (SGD)) were retained for analysis of evolutionary rates, leading to the retention of a set of 256 genes for YTN1 and a set of 138 genes for YTN2. Because the first network contains 85% more transcription factors than the second, we have much more power to detect significant effects using the first network and therefore focus most of our discussion on that dataset. Nevertheless, both datasets yield qualitatively similar results for each of our major conclusions.

Large-scale analyses have shown that multiple genomic variables have an effect on the rate of protein evolution [16,17]. Among them, expression level has been shown to correlate strongly with a gene's evolutionary rate [18-22], leading to a wide debate about the importance of other genomic variables such as essentiality [21-25] and connectivity [7,21,24,26-28]. Therefore, we first examine the separate effects of expression, function and network related variables on rates of transcription factor sequence evolution in turn, and then tease apart their independent effects using a multivariate approach.

**Figure 1:**
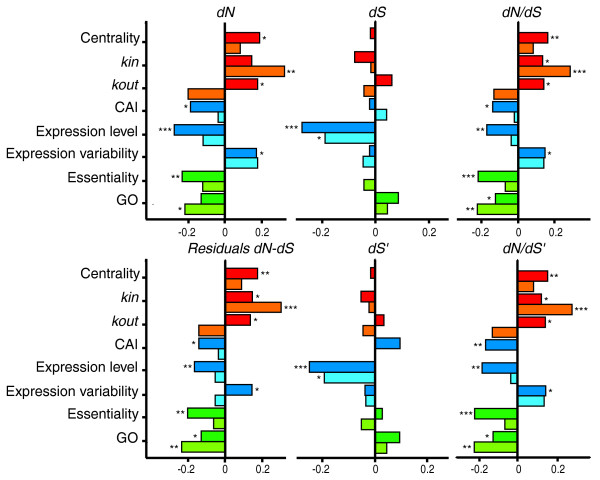
Correlation between expression (blue), function (green), and network topology (red) related variables with evolutionary rates. Darker colors represent results from analyses of YTN1, and lighter colors represent results from analyses of YTN2. Correlations are Spearman's nonparametric ρ. *P < 0.05, **P < 0.01, ***P < 0.001.

As noted in previous studies, expression level has a strong effect on transcription factor sequence evolution (Figure 1), with more highly expressed genes being under stronger purifying selection against both amino acid replacements and synonymous changes, as predicted by the translational robustness hypothesis [20]. Further, essential transcription factors (those having a lethal phenotype in deletion-mutants [29]) also tend to evolve slower than non-essential transcription factors, at least in the network that includes more transcription factors (YTN1: *dN*/*dS*: *t*_249 _= 3.62, *P *< 0.001; Wilcoxon two-sample *P *< 0.0001). Similarly, we find a correlation between protein evolutionary rates and genes' essentiality estimated by the growth rate of deletion strains [19] (Figure 1).

To further investigate the impact of functional constraints on sequence evolution, we used the number of Gene Ontology (GO) terms [30,31] as a proxy for a gene's pleiotropic effects. GO describe a gene's properties and functions by assigning attributes under the categories 'cellular component', 'biological process' and 'molecular function'. There is a correlation between the number of GO terms and essentiality (YTN1: Spearman's ρ = 0.171, *P *= 0.007), indicating that pleiotropy has direct fitness consequences. Accordingly, transcription factors with more GO terms tend to evolve more slowly (Figure 1), presumably because mutations arising in genes with larger pleiotropic effects are more likely to be deleterious and are thus selected against.

Finally, the position of a gene within the network, or its centrality, has a significant influence on its evolutionary rate (Figure 1). Previous studies have determined that central metabolic enzymes and central proteins in the PPI network are under stronger selective constraints and evolve slower [4,6]. In contrast, we find that for the transcriptional network, protein evolution is positively correlated with betweenness, indicating that transcription factors that occupy a more central position in the network tend to evolve faster (Figure 1). Similarly, contrary to metabolic and PPI networks, protein sequence divergence correlates positively with connectivity. However, the relationship between out-degree and evolutionary rate differs between the two network datasets (Figure 1).

### The effect of centrality on protein sequence evolution is independent of other genomic variables

Interpretation of these simple correlation patterns is complicated by the fact that different genetic properties are correlated with one another and so any single correlation between two characteristics might actually be generated by a shared correlation with a third causal element. To correct for this, we examined the relative contribution of function, network and expression-associated constraints on transcription factor evolution using multivariate analysis.

We first used multiple regression analysis with network connectivity and network centrality separately with function and expression-related predictor variables in order to estimate the contribution of each of these elements to variation in evolutionary rates among transcription factors. Consistent with the univariate patterns, our analysis reveals that transcription factors having larger effects on organismal fitness when deleted tend to evolve more slowly than those with lesser fitness effects (Table 1). In the same vein, transcription factors with a larger number of GO terms are subject to stronger functional constraints and tend to evolve more slowly (Table 1). These results indicate that sequence divergence for the yeast transcription factors depends at least in part on the cost of mutations altering protein function and affecting organismal fitness. Among the genomic variables analyzed, only expression level has a strong effect on the rate of synonymous changes (Table 2)

**Table 1:** Multiple regression of genomic variables and protein evolutionary rates

	*dN*		*dN*/*dS*		*dN*/*dS'*		Residuals *dN*-*dS*	
				
Predictor	YTN1	YTN2	YTN1	YTN2	YTN1	YTN2	YTN1	YTN2
Relationships between evolutionary rates and six predictor variables								
Expression level	-0.105	-0.022	-0.043	0.058	-0.051	0.061	-0.038	0.050
Expression variability	-0.047	-0.064	-0.010	-0.015	-0.011	-0.013	-0.019	-0.024
CAI	-0.096	-0.037	0.026	-0.060	-0.088	-0.046	-0.072	-0.055
GO	-0.135*	-0.277^†^	-0.149*	-0.311^‡^	-0.165^†^	-0.313^‡^	-0.168^†^	-0.307^‡^
Essentality	-0.185^†^	-0.104	-0.229^‡^	-0.121	-0.208^‡^	-0.122	-0.181^†^	-0.121
Centrality	0.162^†^	0.199*	0.151*	0.191*	0.164^†^	0.190*	0.164^†^	0.201*
								
Relationships between evolutionary rates and seven predictor variables								
Expression level	-0.107	-0.030	-0.044	0.047	-0.053	0.050	-0.040	0.041
Expression variability	-0.053	-0.084	-0.016	-0.036	-0.020	-0.035	-0.028	-0.043
CAI	-0.111	-0.059	0.010	-0.083	-0.105	-0.069	-0.091	-0.077
GO	-0.113	-0.204*	-0.129*	-0.244^†^	-0.142*	-0.246^†^	-0.145*	-0.237^†^
Essentiality	-0.177^†^	-0.039	-0.222^‡^	-0.060	-0.199^†^	-0.062	-0.173^†^	-0.057
*k*_*in*_	0.139*	0.291^†^	0.132*	0.288^†^	0.148*	0.288^†^	0.152*	0.290^†^
*k*_*out*_	0.042	-0.165	0.032	-0.148	0.037	-0.147	0.031	-0.154

Network, function and expression-related variables have independent effects on the rate of protein evolution. Entries show standardized regression coefficients. *P < 0.05, ^†^*P *< 0.01, ^‡^*P *< 0.001.

**Table 2:** Multiple regression of genomic variables and rates of synonymous changes

	*dS*		*dS'*	
		
Predictor	YTN1	YTN2	YTN1	YTN2
Relationships between evolutionary rates and six predictor variables				
Expression level	-0.216^†^	-0.229*	-0.218^†^	-0.229*
Expression variability	-0.090	-0.137	-0.091	-0.137
CAI	-0.086	0.050	0.046	0.005
GO	0.075	0.051	0.076	0.051
Essentality	-0.041	0.037	-0.041	0.037
Centrality	0.020	0.026	0.020	0.026
				
Relationships between evolutionary rates and seven predictor variables				
Expression level	-0.213^†^	-0.228*	-0.215^†^	-0.229*
Expression variability	-0.083	-0.140	-0.084	-0.140
CAI	-0.077	0.046	0.055	0.001
GO	0.075	0.069	0.076	0.069
Essentiality	-0.039	0.053	-0.040	0.053
*k*_*in*_	-0.014	0.048	-0.014	0.048
*k*_*out*_	0.039	-0.059	0.040	-0.059

Entries show standardized regression coefficients. *P < 0.05, ^†^*P *< 0.01.

We find no significant correlation between in-degree and essentiality (*k*_*in*_: YTN1: Spearman's ρ = -0.082, *P *= 0.2; YTN2: ρ = 0.033, *P *= 0.7), although the relationship between out-degree and essentiality differs between the two datasets (*k*_*out*_: YTN1: Spearman's ρ = -0.071, *P *= 0.26; YTN2: ρ = 0.27, *P *= 0.002). However, when growth rate is measured under different conditions, transcription factors with numerous target genes in YTN2 are not enriched in essential genes [14]. Nevertheless, the correlation between the number of target genes and protein sequence divergence is fairly weak, as multiple regression analysis failed to disentangle the effect of out-degree from the causal effect of other predictor variables (Table 1). Therefore, contrary to the PPI [4,8] and metabolic [5,6] networks, there is no significant correlation between connectivity and essentiality, while in-degree is in fact positively correlated with protein sequence divergence.

Importantly, this analysis also shows that the contribution of network centrality to protein divergence is independent of expression and function-related variables (Table 1). Thus, a striking difference among cellular networks lies in the influence of the position of a gene within the network on its rate of evolution. However, transcription factors that are more central in the network do tend to show higher variability in their expression level under changing conditions (YTN1: Spearman's ρ = 0.178, *P *= 0.004), but centrality is not correlated with expression level (YTN1: Spearman's ρ = 0.006, *P *= 0.924) and essentiality (YTN1: Spearman's ρ = -0.022, *P *= 0.735).

The high degree of correlation among predictor variables has led some to question the use of multiple regression for these types of analyses [21]. We therefore also analyzed these data using principal component regression analysis [21]. For YTN1, the first principal component, composed mostly of contributions from network-related variables, is positively correlated with protein divergence but the second principal component, mostly composed of expression and function-related variables, correlates negatively with substitution rates (Tables 3 and 4). Both principal components explain a similar amount of the total variance in the data, indicating that no single variable dominates the rate of protein evolution for the yeast transcription factor genes. The pattern is more complex for YTN2 because the principle components tend to confound expression and network properties. For instance, the first principle component for YTN2 does not show a significant effect on evolutionary rate, presumably because the positive and negative effects of the network, function, and expression variables are counterbalancing one another (Tables 3 and 4).

**Table 3:** Principal component regression analysis: principal components PC1 to PC4

	PC1		PC2		PC3		PC4	
				
	YTN1	YTN2	YTN1	YTN2	YTN1	YTN2	YTN1	YTN2
Percent variance explained by each PC	27	29	20	19	15	15	12	10
								
Effect of PCs on response variables								
*dN*	0.118^†^	-0.026	-0.172^‡^	0.166*	0.015	0.199^†^	-0.065	0.144
*dS*	-0.001	-0.009	-0.122*	-0.046	-0.161^†^	-0.094	0.073	0.096
*dS'*	-0.014	-0.019	-0.053	-0.030	-0.108	-0.114	0.082	0.078
*dN*/*dS*	0.114^†^	-0.028	-0.113*	0.194^†^	0.106	0.240^†^	-0.123*	0.117
*dN*/*dS'*	0.132^†^	-0.025	-0.169^‡^	0.190^†^	0.064	0.248^†^	-0.106	0.122
Residuals *dN*-*dS*	0.124^†^	-0.024	-0.141^†^	0.189^†^	0.071	0.239^†^	-0.093	0.120
								
Contribution of predictor variables to each PC								
CAI	-0.095	0.228	0.535	-0.356	0.419	0.435	0.069	0.405
Expression level	0.008	0.289	0.515	-0.297	0.154	0.468	-0.596	-0.280
Expression variability	0.290	-0.105	-0.350	0.616	0.454	-0.050	0.132	-0.009
*k*_*in*_	0.538	0.423	0.195	0.413	0.288	0.333	0.061	0.140
*k*_*out*_	0.464	0.453	-0.047	0.052	-0.419	-0.353	-0.023	-0.157
Centrality	0.623	0.564	0.176	0.312	-0.095	-0.010	0.067	0.050
Essentiality	-0.115	0.230	0.415	-0.241	-0.102	-0.506	0.776	0.634
GO	0.011	0.314	0.287	-0.277	-0.562	-0.313	-0.109	-0.556

No single variable dominates the rate of protein evolution. *P < 0.05, ^†^*P *< 0.01, ^‡^*P *< 0.001. PC, principal component.

**Table 4:** Principal component regression analysis: principal components PC5 to PC8

	PC5		PC6		PC7		PC8	
				
	YTN1	YTN2	YTN1	YTN2	YTN1	YTN2	YTN1	YTN2
Percent variance explained by each PC	10	9	7	8	7	8	2	2
								
Effect of PCs on response variables								
*dN*	-0.067	-0.170	-0.127	0.109	-0.144	-0.188	-0.088	-0.213
*dS*	0.055	0.078	-0.043	0.034	-0.131	-0.249	0.011	0.074
*dS'*	0.081	0.050	0.022	0.046	-0.195*	-0.251*	-0.014	-0.073
*dN*/*dS*	-0.045	-0.209*	-0.052	0.098	-0.167*	-0.106	-0.103	-0.147
*dN*/*dS'*	-0.081	-0.201*	-0.117	0.093	-0.114	-0.102	-0.083	-0.143
Residuals *dN*-*dS*	-0.089	-0.203*	-0.119	0.103	-0.108	-0.118	-0.089	-0.200
								
Contribution of predictor variables to each PC								
CAI	0.194	0.624	0.501	-0.279	-0.485	0.027	-0.022	-0.025
Expression level	-0.255	-0.366	0.025	0.178	0.539	0.603	0.001	-0.032
Expression variability	0.400	0.447	0.377	0.098	0.518	0.630	-0.010	-0.020
*k*_*in*_	0.101	-0.060	-0.505	0.328	-0.139	-0.296	0.549	0.567
*k*_*out*_	-0.353	-0.090	0.584	-0.691	-0.062	0.177	0.370	0.354
Centrality	-0.026	-0.086	-0.102	-0.023	-0.083	-0.170	-0.741	-0.738
Essentiality	-0.192	-0.125	-0.022	0.367	0.402	0.277	-0.049	-0.063
GO	0.752	0.492	0.012	0.400	0.121	-0.119	0.098	0.047

No single variable dominates the rate of protein evolution. *P < 0.05. PC, principal component.

To get around these issues, we defined a new set of variables composed of principal components derived separately from the expression, network and function-related variables. Multiple regression analysis on these composite variables shows that each of these causal components has independent effects on the rate of nonsynonymous changes (Table 5). Results from the two network datasets are qualitatively and quantitatively very similar to one another, although particular coefficients from YTN2 tend to be less significant because of reduced power.

**Table 5:** Results of multiple regression analysis on composite variables

	PC1-network		PC1-expression		PC1-function	
			
	YTN1	YTN2	YTN1	YTN2	YTN1	YTN2
Percent of variance explained by PC1	69	65	48	50	54	60
*dN*	0.095*	0.099	-0.112*	-0.007	-0.196^†^	-0.246^†^
*dS*	-0.016	-0.031	-0.170^†^	-0.032	0.065	0.098
*dS'*	-0.016	-0.032	-0.094	-0.058	0.068	0.099
*dN*/*dS*	0.098*	0.112	-0.014	-0.001	-0.249^‡^	-0.295^‡^
*dN*/*dS'*	0.107^†^	0.113	-0.083	0.008	-0.245^‡^	-0.298^‡^
Residuals *dN*-*dS*	0.106*	0.113	-0.061	0.003	-0.229^‡^	-0.289^‡^

Each composite variable is the first principal component of expression (CAI, expression level, expression variability), network (betweenness, *k*_*in*_, *k*_*out*_) and function (GO, essentiality) related variables. *P < 0.05, ^†^*P *< 0.01, ^‡^*P *< 0.001.

In summary, our results on the yeast transcription network and previous work on the yeast metabolic and PPI networks [4-6] show that the structure of cellular networks influences the evolution of proteins within these networks. However, the system-level pattern of selective constraints at individual nodes differ despite the three networks having grossly similar topologies, perhaps in relation with the function and the nature of the network.

## Discussion

Genomic information generated in recent years has not only offered new insights into biological processes at various levels of organization [1-3,32], but has also enabled a shift from studying the evolution of single or few genes to a system-level view of molecular evolution that integrates interactions among genes within their cellular context. A first consequence of this new perspective is the recognition that several factors in addition to protein function control rate divergence in coding sequences [16,17], with expression level having a strong effect [20,21].

A second consequence of this systems molecular evolution perspective is that it yields novel insights into how cellular networks and their components evolve. Previous studies have noted that metabolic enzymes with high degree are no more essential than those with low degree, perhaps because rerouting of metabolic fluxes in highly connected regions circumvents loss of function mutations at a given node [5]. The absence of correlation between connectivity and essentiality observed here may be the consequence of a similar mechanism of genetic robustness achieved through rerouting of information flow through the transcriptional network. This hypothesis is further suggested by a recent study showing that the mean sequence divergence among intermediate regulators between a top regulator and its target gene increases with the number of alternative pathways between the regulator-target gene pair [33].

We obtain qualitatively similar results from our analysis of both representations of the transcriptional network [14,15]. This is especially true if we account for the overall correlation structure among the variables within the network, function, and expression classes (Table 5). Many more transcription factors are represented in the first network, however, which makes it much easier to detect significant evolutionary associations. It is clear, therefore, that completeness of the network will influence conclusions from global analyses such as that conducted here. Nevertheless, the fact that similar results are obtained from different network datasets, which undoubtedly capture different levels of network complexity, suggests that the results presented here are somewhat robust to overall sampling issues.

Our results on the yeast transcription network and previous work on the yeast metabolic and PPI networks [4-6] show that the structure of cellular networks influences selective constraints at individual nodes, but that these system-level constraints differ despite the three cellular networks having similar, although not identical, topological properties [1,13]. These differences may ultimately be due to the nature of the networks and how they function. Highly connected proteins in the PPI and metabolic networks are subject to stronger purifying selection, presumably because of a larger fraction of sites involved in interactions and because of kinetic constraints due to highly used metabolites, respectively [5,7].

In contrast, transcription networks play fundamental roles in regulating cell state during developmental processes and during physiological adjustment to changing environmental conditions [12]. For instance, changes in growth conditions lead *Escherichia coli *to regulate transcript and protein levels to maximize growth rate and maintain stable metabolite levels, whereas when enzymes of the carbon metabolism network are disrupted, system stability is achieved through redundancy and flux rerouting [34]. In eukaryotes other than yeast, transcriptional variability (which might serve as an indicator of environmental sensitivity), rather than expression level *per se*, seems to correlate better with protein divergence [35]. Here, transcription factors that are more central in the network tend to show higher variability in their expression level in changing conditions. At a local scale, expression variability within a regulatory motif also depends on network structure [36]. However, we do not find a significant effect of expression variation on transcription factor evolution (Table 1). The influence of centrality on the rate of protein evolution in the yeast transcription factor network is therefore not a secondary effect of selection acting directly on transcriptional variability. Because central transcription factors have rapid access to many regions of the network and may act to control the flow of information across the network, they may be important components of sensory systems that transduce environmental changes and coordinate the response of the regulatory network. It is possible that the higher level of amino acid change seen in central transcription factors is therefore the result of historical adaptation to changing environmental conditions. An alternative hypothesis is that more central transcription factors are instead more buffered from outside influences and therefore less subject to strong purifying selection.

Although the relationship between centrality and evolutionary rate is somewhat unexpected, examination of the fine scale structure of other networks indicates that this may be a general property of control systems. For example, although highly connected proteins (hubs) in the yeast PPI network evolve slowly [4,7], intermodule hubs (those that display temporal variation in their connections) are more divergent than intramodule hubs (those displaying static patterns of interactions) [37]. Similarly, directional selection has recently been inferred at controlling, branch-point enzymes in four out of five metabolic pathways converging to glucose-6-phosphate in *Drosophila *[38]. Thus, proteins that exert some control in flux distribution, information processing or in connecting various protein complexes may, in general, be the target of adaptation because mutations arising in these proteins have the potential to affect the entire system and may, therefore, be more exposed to natural selection.

## Conclusions

The system-level pattern of evolutionary rates is different from that observed in the protein-protein interaction and metabolic networks: central transcription factors tend to evolve faster. This suggests that the higher nucleotide rate divergence in central transcription factors may result from the role that these proteins play in controlling the flow of information and may be the result of adaptation to changing environmental conditions. The conclusions derived from network level analyses of molecular evolution can clearly vary depending on the functional role played by the components of that network. In the same way that we have shown that the particular function of a network can influence how one interprets the impact of its structure on protein evolution, it is clear that we must begin to link all of these networks (regulatory, protein-protein, and metabolic) together so that the complete nature and consequences of network structure on the function and evolution of biological systems can be examined.

## Materials and methods

We used two distinct datasets of the yeast transcriptional network. The first dataset [15], YTN1, includes 286 transcription factors, 3,369 target genes and 8,372 regulatory interactions. The second dataset [14], YTN2, includes 157 transcription factors, 4,410 target genes and 12,873 regulatory interactions. The two networks were derived from largely independent genetic, biochemical and ChIP-chip experiments. Node statistics, including in-degree (*k*_*in*_), out-degree (*k*_*out*_) and betweenness, were obtained for each dataset using the tYNA platform [39].

Protein sequences of orthologous genes from *Saccharomyces cerevisiae *[40] and *S. paradoxus*, the most closely related species [41] having its genome sequenced [42], were retrieved from the SGD [43], aligned [44], and subsequently used to generate codon-based DNA sequence alignments. Maximum likelihood estimates of the rates of amino acid replacements (*dN*) and synonymous changes (*dS*) were computed in CODEML [45]. In addition, we computed the rate of synonymous changes corrected for selection at silent sites (*dS'*) [46]. We also attempted to correct for the correlation between *dN *and *dS *by using the residuals of the regression between *dN *with *dS *in our analyses.

Essentiality was defined by a lethal phenotype in deletion strains [29]. For a quantitative measure of a gene's essentiality we used growth rates measured in deletion mutants [19]. The number of GO terms [30,31] used as a proxy for a gene's pleiotropic effect was obtained from the SGD. Protein and mRNA abundance have been used as estimates of gene expression in studies of evolutionary rates in yeast [18-22,24,25,37]. We obtained protein [47] and mRNA [48] abundance from the literature. However, in our sample faster evolving genes are more likely to be missing from the mRNA abundance (YTN1: N = 206; YTN2: N = 108) and protein abundance (YTN1: N = 195; YTN2: N = 96) datasets, leading to an obvious bias (YTN1: protein abundance: *dN/dS*: Wilcoxon two-sample *P *= 0.004; mRNA abundance: *dN/dS*: Wilcoxon two-sample *P *= 0.04; YTN2: protein abundance: *dN*: Wilcoxon two-sample *P *= 0.03; mRNA abundance: *dN/dS*: Wilcoxon two-sample *P *= 0.06). Nevertheless, the translational robustness hypothesis suggests that the frequency of translation events is a better indicator of evolutionary rate than the number of proteins per cell [20]. Therefore, we used the codon adaptation index (CAI) [49], which measures synonymous codon usage bias and correlates with mRNA abundance [50], as well as direct measures of expression level, as substitutes for other abundance measures. CAI was computed [51] using the reference gene set defined by Carbone *et al*. [52]. Expression level is the average level of expression across 198 microarrays from a wide range of conditions [35]. Expression variation is measured by the coefficient of variation defined as the mean over the standard deviation [35].

Statistical analyses were performed using JMP 4.0.4 (SAS Institute Inc., Cary, NC, USA). The number of GO terms and *k*_*out *_were natural-logarithmic transformed to approximate a normal distribution. One unit was added to betweenness and *k*_*in*_, as well as *k*_*out *_in YTN2, prior to the natural logarithmic transformation because of null values for these variables. All variables, including predictor and response variables, were standardized to a mean of 0 and 1 standard deviation unit. In addition to Spearman's rank correlations and multiple regression analysis, we also performed principal component regression analysis, first using single predictor variables together and then by defining a new set of principal components separately from the expression, network and function-related variables. These composite variables were obtained from the first principal component of expression (CAI, expression level, expression variability), network (betweenness, *k*_*in*_, *k*_*out*_) and function (GO, essentiality) related variables. Principal component analyses were performed on correlations.

## Abbreviations

CAI: codon adaptation index; *dN*: rate of nonsynonymous changes; *dS*: rate of synonymous changes; *dS'*: rate of synonymous changes corrected for selection at silent sites; GO: Gene Ontology; *k*_*in*_: in-degree; *k*_*out*_: out-degree; PPI: protein-protein interaction; SGD: *Saccharomyces *Genome Database.

## Authors' contributions

RJ designed the study and collected the data. RJ and PCP analyzed the data and wrote the paper.
